# Seasonal and Time-of-Day Variations in Ambulance-Transported Emergency Department Diagnoses: A One-Year Observational Study

**DOI:** 10.3390/healthcare14152245

**Published:** 2026-07-23

**Authors:** Fatih Mehmet Sari, Yasin Bilgin, Mert Kilic, Erdem Yakup Cimen, Abdullah Emre Yurttutan, Arif Onur Eden

**Affiliations:** Department of Emergency, Mengucek Gazi Training and Research Hospital, Faculty of Medicine, Erzincan Binali Yildirim University, Erzincan 24100, Türkiye; fatih.sari@erzincan.edu.tr (F.M.S.); mertkilic@gmail.com (M.K.); erdem.cimen@erzincan.edu.tr (E.Y.C.); emre.yurttutan@erzincan.edu.tr (A.E.Y.); aeden@erzincan.edu.tr (A.O.E.)

**Keywords:** emergency medical services, ambulance transport, time-of-day variation, seasonal variation, emergency department, prehospital care, trauma

## Abstract

**Highlights:**

**What are the main findings?**

- Ambulance-transported emergency department diagnoses showed significant seasonal and time-of-day variation across major diagnostic categories.
- Trauma presentations peaked during summer, respiratory diseases were most frequent during winter and psychiatric presentations predominated during the evening hours.

**What are the implications of the main findings?**

- Recognition of temporal diagnostic patterns may support more efficient emergency department staffing, EMS resource allocation, and operational planning.
- These findings may contribute to evidence-based planning of emergency medical services according to seasonal and time-of-day variations in patient presentations.

**Abstract:**

**Background/Objectives**: Ambulance-transported patients represent an important subgroup of emergency department (ED) admissions and may demonstrate distinct temporal presentation patterns. However, studies simultaneously evaluating broad diagnostic categories according to both time-of-day and seasonal variations among ambulance-transported patients remain limited. **Methods**: This retrospective observational study included 37,188 patients transported to the emergency department of a tertiary university hospital by ambulance between December 1, 2024, and November 30, 2025. Patients were categorized into twelve major diagnostic groups according to ICD-10-based clinical diagnoses. Temporal analyses were performed according to seasons and time-of-day intervals (nighttime, daytime, and evening). Associations between diagnostic categories and temporal variables were evaluated using chi-square analysis. **Results**: Trauma was the most common diagnostic category (19.9%), followed by cardiovascular (16.3%), gastrointestinal (10.3%), and respiratory diseases (10.1%). Significant associations were observed between diagnostic categories and both seasonal distribution (χ^2^ = 446.482, df = 33, *p* < 0.001) and time of presentation (χ^2^ = 642.883, df = 22, *p* < 0.001). Respiratory diseases were most frequent during winter (32.7%), whereas trauma-related admissions peaked during summer (32.4%). Psychiatric presentations predominantly occurred during evening hours (53.2%), while gastrointestinal diseases demonstrated the highest proportion of nighttime admissions (24.2%). Trauma-related admissions were more common among males (63.6%), whereas neurological, gastrointestinal, metabolic, and psychiatric presentations were slightly more frequent among females. The mean age of the cohort was 49.02 ± 25.12 years. **Conclusions**: Ambulance-transported emergency department patients demonstrate significant seasonal and time-of-day variations across major diagnostic categories. Awareness of these temporal patterns may contribute to improved emergency department organization, ambulance deployment, staffing strategies, and healthcare resource management.

## 1. Introduction

Emergency medical services (EMS) constitute one of the fundamental components of modern healthcare systems by enabling the rapid assessment, stabilization, and transportation of patients requiring urgent medical care. Efficient prehospital systems improve timely access to emergency care, facilitate transport to appropriate healthcare facilities, and play a critical role in reducing preventable morbidity and mortality. In Türkiye, emergency medical services are delivered through a nationwide, publicly funded 112 Emergency Call Center system that provides free ambulance services twenty-four hours a day across both urban and rural regions. As the primary gateway to emergency care, the 112 system has become an essential component of the national healthcare infrastructure, and increasing ambulance utilization has important implications for emergency department (ED) organization, workforce planning, and healthcare resource allocation [1,2,3].

Temporal variation is a well-recognized characteristic of emergency healthcare utilization. Numerous studies have demonstrated that both seasonal and time-of-day factors influence the incidence and distribution of acute diseases as well as patterns of ED attendance. Cardiovascular and cerebrovascular emergencies frequently exhibit time-dependent variation, whereas respiratory diseases and infectious illnesses increase during colder months [4,5,6]. In contrast, trauma-related presentations are generally more common during warmer seasons because of increased outdoor activities, travel, and occupational exposure [7]. Understanding these temporal trends is particularly important for EMS systems because changes in diagnostic profiles directly affect ambulance demand, workforce planning, ED preparedness, and resource allocation.

Patients transported by ambulance differ from the general ED population because they have already undergone prehospital evaluation and require EMS resources before hospital arrival [1]. Consequently, ambulance-transported patients represent a distinct epidemiological population whose diagnostic distribution may not fully reflect that of all ED attendees. Evaluating temporal changes specifically within this patient group may therefore provide valuable information for ambulance deployment, staffing strategies, operational planning, and emergency department preparedness [8].

Although the temporal distribution of ED visits has been widely investigated, previous studies have primarily focused on specific diseases, selected patient populations or limited clinical variables rather than the overall diagnostic profile of ambulance-transported patients [9,10]. Furthermore, although several studies have investigated seasonal or time-of-day variation in ED presentations or ambulance demand, these temporal dimensions have generally been evaluated separately, and comprehensive analyses integrating both across broad diagnostic categories remain limited [11,12]. To the best of our knowledge, published evidence from high-volume tertiary emergency departments in Türkiye remains limited. Addressing this knowledge gap may improve understanding of temporal changes in ambulance-transported patient profiles and support evidence-based EMS planning, emergency department preparedness, and healthcare resource allocation. Therefore, further studies comprehensively evaluating seasonal and time-of-day variations across major diagnostic groups among ambulance-transported ED patients are warranted [13,14,15]. Findings from such studies may provide valuable evidence for optimizing EMS deployment strategies and emergency department preparedness within nationwide emergency care systems.

The aim of this study was to investigate seasonal and time-of-day variations in major diagnostic categories among patients transported to the emergency department by ambulance over a one-year period. We further sought to evaluate how these temporal patterns may contribute to emergency department preparedness, EMS planning, and healthcare resource allocation. We hypothesized that significant seasonal and time-of-day variation exists across major diagnostic categories among ambulance-transported emergency department patients.

## 2. Materials and Methods

### 2.1. Study Design and Setting

This retrospective observational study was conducted in the emergency department of Erzincan Binali Yildirim University Mengücek Gazi Training and Research Hospital, a high-volume tertiary referral center located in Eastern Türkiye. The ED provides 24 h emergency care for a broad spectrum of medical, surgical, and traumatic conditions and serves Erzincan Province and neighboring regions. Patient data were obtained from the 112 EMS Command and Control Center database. The study included all eligible ambulance-transported ED admissions recorded between 1 December 2024 and 30 November 2025.

### 2.2. Study Population

A total of 43,238 EMS records were initially identified for the study period. After applying the predefined inclusion and exclusion criteria, the final analytical cohort consisted of 37,188 ambulance-transported emergency department presentations of patients of all age groups. Because all eligible ambulance-transported ED admissions during the study period were included, no sampling procedure was performed. No age-based exclusion criteria were applied, and both pediatric and adult patients were analyzed within the same study cohort. Each ambulance transport was considered an individual study case. Therefore, if the same patient was transported to the ED more than once during the study period, each admission was analyzed separately.

### 2.3. Inclusion and Exclusion Criteria

All patients transported to the ED by the regional 112 EMS during the study period were eligible for inclusion.

The following cases were excluded:

- Patients evaluated but not transported to the hospital (patients left at the scene);
- Patients transferred from the hospital to home or to another healthcare facility;
- Patients transported for outpatient examination or imaging purposes;
- Patients with incomplete or insufficient demographic, temporal, or diagnostic information.

### 2.4. Data Collection and Management

The following variables were extracted from the EMS database:

- Age;
- Sex;
- Date of ED admission;
- Time of ED admission;
- Admission diagnosis.

The EMS database is routinely completed by trained EMS personnel immediately after patient transport as part of standard clinical documentation.

Before statistical analysis, the dataset was reviewed to identify duplicate, incomplete, and inconsistent records. Cases meeting the predefined exclusion criteria were removed. No patient identifiers were available to the investigators during data extraction or analysis, and all analyses were performed using anonymized administrative EMS records to ensure patient confidentiality.

Records with incomplete demographic, temporal, or diagnostic information were excluded before analysis; therefore, the final analytical dataset contained no missing values for the analyzed variables.

### 2.5. Diagnostic Classification

Patients were categorized into twelve major diagnostic groups according to their primary admission diagnoses and corresponding ICD-10 codes:

- Infectious diseases (A00–B99);
- Cardiovascular diseases (I00–I99, I26);
- Neurological diseases (G00–G99, I60–I69);
- Respiratory diseases (J00–J99);
- Gastrointestinal and hepatobiliary diseases (K00–K93);
- Endocrine and metabolic diseases (E00–E90);
- Renal and genitourinary diseases (N00–N99);
- Trauma and injury (S00–T98);
- Psychiatric diseases (F00–F99);
- Musculoskeletal and dermatological diseases (M00–M99, L00–L99);
- Obstetric and gynecologic diseases (O00–O99);
- Other diseases, including symptom-based diagnoses, congenital disorders, perinatal conditions, and administrative codes (R00–R99, Z00–Z99, H00–H95, Q00–Q99, P00–P96).

Diagnostic classifications were based on the admission diagnosis recorded in the 112 EMS case forms. Cerebrovascular diseases (I60–I69) were classified under neurological diseases, whereas pulmonary embolism (I26) was categorized under cardiovascular diseases because of its vascular pathophysiology. Definitive final hospital diagnoses were not independently verified because the study was designed to evaluate temporal patterns using prehospital admission diagnoses recorded by EMS personnel.

### 2.6. Temporal Classification

Patients were classified according to both seasonal categories and time-of-day intervals based on their admission times.

For seasonal analyses, admissions were classified according to the calendar month of presentation as follows:

- Winter (December–February);
- Spring (March–May);
- Summer (June–August);
- Autumn (September–November).

Time-of-day categories were defined according to the routine operational shift structure used by the regional EMS system:

- Nighttime (00:00–07:59);
- Daytime (08:00–15:59);
- Evening (16:00–23:59).

### 2.7. Statistical Analysis

Statistical analyses were performed using IBM SPSS Statistics for Windows, Version 26.0 (IBM Corp., Armonk, NY, USA).

Categorical variables were presented as numbers and percentages (%), whereas continuous variables were expressed as mean ± standard deviation.

Associations between diagnostic categories and seasonal or time-of-day variables were evaluated using the Pearson’s chi-square test. Comparisons of continuous variables between two groups were performed using the independent-samples *t*-test or the Mann–Whitney U test, depending on data distribution. Comparisons among multiple groups were performed using one-way analysis of variance (ANOVA) or the Kruskal–Wallis test, as appropriate. A two-sided *p* value < 0.05 was considered statistically significant.

## 3. Results

A total of 37,188 ambulance-transported emergency department presentations were included in the study. Males represented 53.9% of the cohort. Presentations were most frequent during summer (29.0%) and least frequent during winter (22.2%). According to time of presentation, evening (41.7%) and daytime (40.7%) admissions predominated, whereas nighttime admissions accounted for 17.6%. The most common diagnostic category was trauma (19.9%), followed by cardiovascular (16.3%), gastrointestinal (10.3%), and respiratory disease presentations (10.1%) (Table 1).

A significant association was observed between diagnostic categories and seasons (χ^2^ = 446.482, df = 33, *p* < 0.001). Respiratory diseases were most frequently encountered during winter (32.7%), whereas trauma-related admissions peaked during summer (32.4%). Gastrointestinal diseases showed increased frequencies during summer (27.8%) and autumn (27.4%). Psychiatric presentations demonstrated a relatively balanced seasonal distribution throughout the year (Table 2).

Diagnostic categories also differed significantly according to time of presentation (χ^2^ = 642.883, df = 22, *p* < 0.001). Psychiatric presentations occurred predominantly during evening hours (53.2%), whereas trauma-related admissions were more common during daytime and evening periods (44.1% each). Gastrointestinal diseases showed the highest proportion of nighttime admissions among the major diagnostic groups (24.2%) (Table 3).

A significant association was found between diagnostic categories and sex (χ^2^ = 1013.530, df = 11, *p* < 0.001). Trauma-related admissions were predominantly observed among males (63.6%), whereas neurological (52.6%), gastrointestinal (53.4%), metabolic (51.3%), and psychiatric (52.0%) presentations were slightly more frequent among females (Table 4).

The mean age of the cohort was 49.02 ± 25.12 years. The highest mean age was observed in the other diagnostic category (60.30 ± 24.77 years), followed by cardiovascular (57.75 ± 21.12 years), respiratory (57.39 ± 25.49 years), and metabolic (57.39 ± 22.97 years) presentations. The lowest mean ages were observed in gynecologic and obstetric (28.79 ± 10.68 years), psychiatric (36.17 ± 16.16 years), and trauma-related admissions (39.44 ± 24.03 years) (Table 5). Mean age differed significantly among the diagnostic categories (one-way ANOVA, F(11, 37,176) = 448.43, *p* < 0.001). Post hoc Games–Howell analysis demonstrated significant differences between most diagnostic categories.

**Figure 1:**
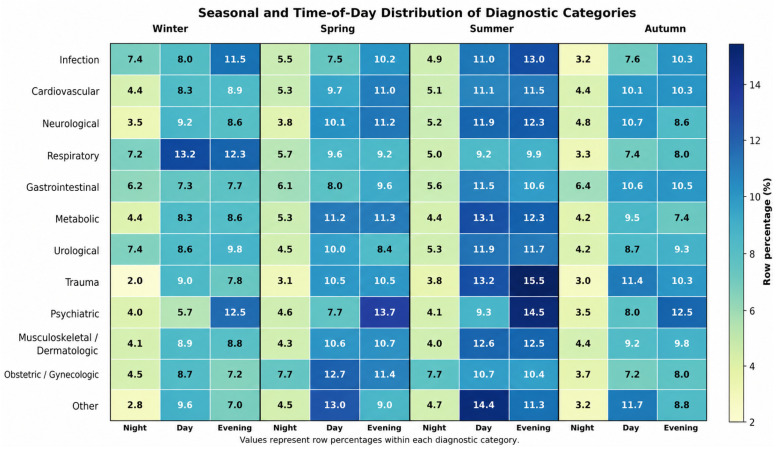
Heatmap showing the seasonal and time-of-day distribution of major diagnostic categories among ambulance-transported emergency department patients. Values represent row percentages within each diagnostic category, with darker colors indicating higher relative frequencies.

Temporal variations in diagnostic categories according to season and time of presentation are illustrated in Figure 1. Trauma-related admissions demonstrated a marked increase during summer evenings (15.5%). Respiratory presentations were more frequent during winter daytime and evening hours, whereas psychiatric admissions consistently predominated during evening periods across all seasons. Gastrointestinal diagnoses were relatively more common during summer and autumn evenings. The heatmap visualization demonstrated distinct seasonal and time-of-day patterns among several major diagnostic categories (Figure 1).

## 4. Discussion

In this study, major diagnostic categories among ambulance-transported emergency department patients demonstrated significant seasonal and time-of-day variations. Trauma was the most common reason for ambulance transport and showed a marked increase during the summer months and evening hours. In contrast, respiratory diseases occurred more frequently during winter, whereas psychiatric presentations predominated during the evening. Gastrointestinal diseases were more common during the summer and autumn seasons. In addition, significant differences in age and sex distributions were observed among several diagnostic categories, indicating that both temporal and demographic factors may influence the epidemiological characteristics of ambulance-transported patients. These findings demonstrate that ambulance-transported patients represent a distinct patient population with dynamic temporal patterns that may have important implications for EMS planning and emergency department preparedness.

Compared with the overall emergency department population, ambulance-transported patients constitute a distinct subgroup requiring prehospital assessment and EMS resources before hospital arrival. Therefore, understanding temporal changes in this population is important not only from an epidemiological perspective but also for optimizing ambulance deployment, emergency department preparedness, and healthcare resource allocation [1]. In our study, trauma and cardiovascular diseases were the most common diagnostic categories, reflecting the prominent role of the EMS system in transporting patients requiring urgent medical assessment and treatment. Furthermore, the observed seasonal and time-of-day patterns suggest predictable fluctuations in ambulance-transported patient profiles that may support more effective staffing strategies and EMS resource planning.

Trauma was the most common diagnostic category among ambulance-transported emergency department patients in our study and demonstrated a marked increase during the summer months. Trauma presentations were also concentrated during evening hours. This pattern may be associated with increased outdoor activities, higher traffic density, agricultural activities, and seasonal population movement during the summer months. In Eastern Türkiye, particularly in Erzincan, seasonal agricultural work and associated rural mobility intensify during the summer months [16], which may partly contribute to the observed increase in trauma-related ambulance transports. Previous studies have similarly reported that traumatic injuries increase during warm months because of greater outdoor exposure, increased mobility, and seasonal occupational activities [7]. The significantly higher proportion of male patients within the trauma group in our study is also consistent with the literature and supports previous evidence that males are more frequently exposed to traumatic injuries [17,18].

The increased frequency of cardiovascular and respiratory diseases during winter was one of the notable findings of our study. Cold weather conditions are known to trigger both cardiovascular and respiratory diseases through mechanisms such as increased sympathetic activity, vasoconstriction, elevated blood pressure, and increased respiratory tract infections [5,6]. In particular, the marked increase in respiratory diseases during winter may be associated with the higher incidence of respiratory tract infections during colder months [19]. In Eastern Türkiye, prolonged cold weather, increased time spent indoors, and seasonal heating practices may further contribute to the higher burden of respiratory diseases during winter. These regional environmental conditions may facilitate the transmission of respiratory infections and increase exacerbations of chronic respiratory diseases. Similarly, the higher frequency of cardiovascular diseases among older age groups is consistent with previous epidemiological data [20]. The substantial proportion of cardiovascular and respiratory diseases among ambulance-transported patients highlights the importance of ensuring adequate EMS capacity during winter months, when time-critical medical emergencies become more frequent.

The predominance of psychiatric presentations during evening hours was another important observation of our study. Possible explanations include accumulated psychosocial stress throughout the day, increased alcohol and substance use, social conflicts, and reduced access to mental health services outside regular working hours. Although biological time-of-day variation may contribute to the observed pattern [21], organizational and social factors are also likely to influence the increased frequency of psychiatric ambulance transports during the evening hours. In addition, ambulance utilization for psychiatric emergencies may be influenced by limited access to outpatient mental health services during off-hours and by differences in help-seeking behaviors and ambulance-calling practices. The considerable proportion of psychiatric presentations within the ambulance system demonstrates that prehospital emergency healthcare services carry a substantial burden not only for medical and traumatic conditions but also for mental health crises.

The higher frequency of neurological diseases among older patients and their predominance during daytime hours support the close association of cerebrovascular events with age-related vascular risk factors, hypertension, atherosclerosis, and cardiovascular comorbidities [20]. Previous studies have also reported that stroke and other acute neurological events exhibit time-of-day variation, with a higher incidence during the early daytime hours, possibly because of sympathetic activation and blood pressure fluctuations [4]. Our findings are consistent with these observations, suggesting that similar temporal patterns are also present among ambulance-transported emergency department patients.

The seasonal distribution of gastrointestinal and infectious diseases further supports the influence of environmental factors on emergency department presentations. In our study, infectious diseases were most frequently observed during summer, whereas respiratory diseases predominated during winter. The winter predominance of respiratory diseases is consistent with the increased incidence of respiratory tract infections during colder months [22]. In contrast, the relatively higher frequency of gastrointestinal diseases during summer and autumn may be explained by increased heat exposure, food- and waterborne infections, dehydration, and inadequate food storage conditions during warmer months [23]. In Eastern Türkiye, seasonal agricultural activities, increased outdoor work, and population mobility during the summer months may further contribute to these temporal patterns [16]. Previous studies have similarly demonstrated that seasonal environmental conditions influence the incidence of infectious and gastrointestinal diseases, supporting the temporal patterns observed in our study [24].

The age and sex distributions observed in our study were generally consistent with previous epidemiological findings. Younger male patients predominated in the trauma group, whereas cardiovascular and respiratory diseases were more frequently observed among older adults, reflecting the well-established relationship between chronic disease burden and advancing age [17,18,20]. Likewise, the higher frequency of neurological diseases during daytime hours was consistent with previous reports describing time-of-day variation in acute cerebrovascular events [4]. Overall, these demographic findings further support the consistency of the diagnostic patterns observed among ambulance-transported ED patients.

The marked seasonal and time-of-day variations identified in our study have important clinical implications for emergency department and EMS organization. The concentration of specific diagnostic groups during particular times of day and seasons may contribute to more effective staffing strategies, shift organization, ambulance deployment, and demand forecasting. In particular, the increased frequency of trauma cases during specific periods may necessitate reinforcement of trauma teams and resources during peak hours. Similarly, the seasonal increase in cardiovascular and respiratory diseases highlights the importance of additional preparedness within emergency departments and EMS systems during winter months. The observed temporal patterns may also support the development of season-specific public health interventions, including injury prevention initiatives during summer and respiratory disease awareness campaigns during winter. These findings may also support the development of season-specific prevention strategies and optimization of EMS dispatch protocols. Accordingly, understanding temporal patterns among ambulance-transported patient populations may provide valuable guidance for emergency department crowd management, EMS operational planning, and optimization of healthcare resources.

This study has several limitations. First, because of its retrospective single-center design, the generalizability of the findings may be limited. Second, the analyses were based on routinely collected administrative EMS records, which were not originally designed for research purposes. Diagnostic classifications were based on admission diagnoses documented in 112 case forms, and definitive final hospital diagnoses were not independently verified. Therefore, some cases may have been classified into different diagnostic categories, particularly for conditions with overlapping clinical presentations such as cardiovascular, neurological, metabolic, and psychiatric disorders. In particular, symptom-based presentations (R00–R99), Z-codes (Z00–Z99), congenital diseases (Q00–Q99), perinatal conditions (P00–P96), and several other non-specific ICD groups were categorized under the “other” group, which may have contributed to the heterogeneous nature of this category. Furthermore, because only ambulance-transported ED patients were included, the findings may not be generalizable to the overall emergency department population. In addition, the study did not include population-adjusted incidence rates, clinical severity indicators, triage categories, patient outcomes, or mortality analyses, limiting causal inference and clinical interpretation of the observed temporal patterns. Nevertheless, the large sample size, uninterrupted one-year dataset, and exclusive focus on ambulance-transported patient populations constitute important strengths of the study. Furthermore, data obtained from a high-volume tertiary referral center provide valuable real-world evidence regarding temporal patterns in emergency medical services.

Future multicenter prospective studies incorporating final hospital diagnoses, clinical severity indicators, and patient outcomes are warranted to validate the temporal patterns observed in the present study and to determine whether these patterns are associated with disease severity, clinical outcomes, and more effective EMS planning.

## 5. Conclusions

Major diagnostic categories among ambulance-transported emergency department patients demonstrated significant seasonal and time-of-day variations. Trauma, cardiovascular, respiratory, gastrointestinal, neurological, and psychiatric diseases showed distinct temporal distribution patterns according to season and time of day. Recognition of these temporal variations may support evidence-based emergency department preparedness, EMS operational planning, ambulance deployment, staffing strategies, and more efficient allocation of healthcare resources. Although the findings should be interpreted within the limitations of a retrospective single-center study based on prehospital admission diagnoses, they provide valuable real-world epidemiological evidence that may inform future EMS organization and emergency healthcare planning.

## Figures and Tables

**Table 1:** Descriptive characteristics of the study population.

Variable	Category	*n* (%)
Sex	Male	20,047 (53.9)
	Female	17,141 (46.1)
Season	Winter	8271 (22.2)
	Spring	9324 (25.1)
	Summer	10,775 (29.0)
	Autumn	8818 (23.7)
Time of presentation	Night	6561 (17.6)
	Daytime	15,131 (40.7)
	Evening	15,496 (41.7)
Diagnostic category	Infection	1728 (4.6)
	Cardiovascular	6064 (16.3)
	Neurological	2793 (7.5)
	Respiratory	3766 (10.1)
	Gastrointestinal	3827 (10.3)
	Metabolic	618 (1.7)
	Urological	1018 (2.7)
	Trauma	7417 (19.9)
	Psychiatric	2506 (6.7)
	Musculoskeletal/Dermatologic	2922 (7.9)
	Gynecologic/Obstetric	402 (1.1)
	Other	4127 (11.1)

Note: Percentages were calculated using the total study population as denominator.

**Table 2:** Seasonal distribution of diagnostic categories.

Diagnosis Group	Winter *n* (%)	Spring *n* (%)	Summer *n* (%)	Autumn *n* (%)	Total
Infection	465 (26.9)	400 (23.1)	498 (28.8)	365 (21.1)	1728
Cardiovascular	1304 (21.5)	1579 (26.0)	1678 (27.7)	1503 (24.8)	6064
Neurological	596 (21.3)	703 (25.2)	821 (29.4)	673 (24.1)	2793
Respiratory	1233 (32.7)	921 (24.5)	906 (24.1)	706 (18.7)	3766
Gastrointestinal	810 (21.2)	906 (23.7)	1063 (27.8)	1048 (27.4)	3827
Metabolic	131 (21.2)	172 (27.8)	184 (29.8)	131 (21.2)	618
Urological	263 (25.8)	234 (23.0)	294 (28.9)	227 (22.3)	1018
Trauma	1396 (18.8)	1786 (24.1)	2406 (32.4)	1829 (24.7)	7417
Psychiatric	554 (22.1)	652 (26.0)	699 (27.9)	601 (24.0)	2506
Musculoskeletal/ Dermatologic	636 (21.8)	750 (25.7)	853 (29.2)	683 (23.4)	2922
Gynecologic/Obstetric	82 (20.4)	128 (31.8)	116 (28.9)	76 (18.9)	402
Other	801 (19.4)	1093 (26.5)	1257 (30.5)	976 (23.6)	4127

Note: Percentages are row percentages. Pearson chi-square test: χ^2^ = 446.482, df = 33, *p* < 0.001.

**Table 3:** Distribution of diagnostic categories according to time of presentation.

Diagnosis Group	Night *n* (%)	Daytime *n* (%)	Evening *n* (%)	Total
Infection	363 (21.0)	589 (34.1)	776 (44.9)	1728
Cardiovascular	1159 (19.1)	2378 (39.2)	2527 (41.7)	6064
Neurological	484 (17.3)	1169 (41.9)	1140 (40.8)	2793
Respiratory	798 (21.2)	1487 (39.5)	1481 (39.3)	3766
Gastrointestinal	928 (24.2)	1432 (37.4)	1467 (38.3)	3827
Metabolic	113 (18.3)	260 (42.1)	245 (39.6)	618
Urological	218 (21.4)	400 (39.3)	400 (39.3)	1018
Trauma	878 (11.8)	3271 (44.1)	3268 (44.1)	7417
Psychiatric	403 (16.1)	771 (30.8)	1332 (53.2)	2506
Musculoskeletal/ Dermatologic	492 (16.8)	1207 (41.3)	1223 (41.9)	2922
Gynecologic/Obstetric	95 (23.6)	158 (39.3)	149 (37.1)	402
Other	630 (15.3)	2009 (48.7)	1488 (36.1)	4127

Note: Percentages are row percentages. Pearson chi-square test: χ^2^ = 642.883, df = 22, *p* < 0.001.

**Table 4:** Distribution of diagnostic categories according to sex.

Diagnosis Group	Male *n* (%)	Female *n* (%)	Total
Infection	957 (55.4)	771 (44.6)	1728
Cardiovascular	3251 (53.6)	2813 (46.4)	6064
Neurological	1325 (47.4)	1468 (52.6)	2793
Respiratory	2132 (56.6)	1634 (43.4)	3766
Gastrointestinal	1785 (46.6)	2042 (53.4)	3827
Metabolic	301 (48.7)	317 (51.3)	618
Urological	564 (55.4)	454 (44.6)	1018
Trauma	4715 (63.6)	2702 (36.4)	7417
Psychiatric	1202 (48.0)	1304 (52.0)	2506
Musculoskeletal/ Dermatologic	1766 (60.4)	1156 (39.6)	2922
Gynecologic/Obstetric	0 (0.0)	402 (100.0)	402
Other	2049 (49.6)	2078 (50.4)	4127

Note: Percentages are row percentages. Pearson chi-square test: χ^2^ = 1013.530, df = 11, *p* < 0.001.

**Table 5:** Age distribution according to diagnostic categories.

Diagnosis Group	Age, Mean ± SD	N
Infection	43.51 ± 30.32	1728
Cardiovascular	57.75 ± 21.12	6064
Neurological	51.71 ± 23.29	2793
Respiratory	57.39 ± 25.49	3766
Gastrointestinal	46.72 ± 25.31	3827
Metabolic	57.39 ± 22.97	618
Urological	51.80 ± 24.95	1018
Trauma	39.44 ± 24.03	7417
Psychiatric	36.17 ± 16.16	2506
Musculoskeletal/ Dermatologic	43.30 ± 22.97	2922
Gynecologic/Obstetric	28.79 ± 10.68	402
Other	60.30 ± 24.77	4127

Note: Mean age differed significantly among diagnostic categories (one-way ANOVA, F(11, 37,176) = 448.43, *p* < 0.001). Post hoc comparisons were performed using the Games–Howell test.

## Data Availability

The datasets used and/or analyzed during the current study are not publicly available because of restrictions under the Turkish Personal Data Protection Law. However, anonymized data may be made available from the corresponding author upon reasonable request.

## Abbreviations

The following abbreviations are used in this manuscript:

ED	Emergency Department
EMS	Emergency Medical Services
